# Supplementary material for: MetaRibo-Seq measures translation in microbiomes
Source: Nat Commun. 2020 Jun 29;11:3268. doi: 10.1038/s41467-020-17081-z (PMC7324362; doi:10.1038/s41467-020-17081-z)
Supplement: Supplementary file 10 — Supplementary Data 7 [file 41467_2020_17081_MOESM10_ESM.zip › File2/Confidence_High_Taxonomy/57073_out.krona.html]

Javascript must be enabled to view this page.

members
magnitude
magnitudeUnassigned
count
unassigned
taxon
rank

57073\_out

159

159
superkingdom
2

phylum
976
159

159
200643
class

171549
order
159

159
family
1853231

genus
283168
159

152

SRS011084\_contig\_number\_30292SRS011134\_contig\_number\_34029SRS011239\_contig\_number\_19907SRS011302\_contig\_number\_25426SRS012849\_contig\_number\_16077SRS012969\_contig\_number\_3287SRS013098\_contig\_number\_37024SRS013476\_contig\_number\_29216SRS013521\_contig\_number\_6483SRS013800\_contig\_number\_contig-100\_890.94565SRS014412\_contig\_number\_10677SRS014459\_contig\_number\_2781SRS015065\_contig\_number\_35849SRS015431\_contig\_number\_43737SRS015663\_contig\_number\_25871SRS015782\_contig\_number\_34768SRS015890\_contig\_number\_8402SRS016056\_contig\_number\_3022SRS016095\_contig\_number\_25954SRS016335\_contig\_number\_48415SRS016954\_contig\_number\_contig-100\_243.135792SRS017247\_contig\_number\_contig-100\_185.66330SRS017433\_contig\_number\_contig-100\_13.100734SRS017521\_contig\_number\_38295SRS017821\_contig\_number\_27170SRS018133\_contig\_number\_9079SRS018623\_contig\_number\_20059SRS018817\_contig\_number\_12729SRS018836\_contig\_number\_26886SRS018888\_contig\_number\_7597SRS018984\_contig\_number\_19167SRS019068\_contig\_number\_74415SRS019161\_contig\_number\_32087SRS019397\_contig\_number\_3587SRS019445\_contig\_number\_30703SRS019601\_contig\_number\_11418SRS019787\_contig\_number\_contig-100\_1393.55146SRS019910\_contig\_number\_14766SRS020233\_contig\_number\_50271SRS020271\_contig\_number\_2603SRS020328\_contig\_number\_12924SRS021219\_contig\_number\_10858SRS021484\_contig\_number\_16363SRS022137\_contig\_number\_17441SRS022609\_contig\_number\_37240SRS023526\_contig\_number\_29649SRS023715\_contig\_number\_14613SRS024075\_contig\_number\_10911SRS024132\_contig\_number\_29352SRS024435\_contig\_number\_33617SRS024492\_contig\_number\_10177SRS043001\_contig\_number\_1942SRS043701\_contig\_number\_2211SRS043768\_contig\_number\_27175SRS043841\_contig\_number\_8048SRS044535\_contig\_number\_17738SRS045195\_contig\_number\_contig-100\_213.214SRS045645\_contig\_number\_26589SRS046068\_contig\_number\_contig-100\_448.35194SRS046369\_contig\_number\_13617SRS046502\_contig\_number\_1153SRS046717\_contig\_number\_6734SRS047014\_contig\_number\_32263SRS047044\_contig\_number\_15581SRS048164\_contig\_number\_12934SRS048870\_contig\_number\_7912SRS049402\_contig\_number\_6906SRS049446\_contig\_number\_13218SRS049773\_contig\_number\_2853SRS049900\_contig\_number\_19677SRS049995\_contig\_number\_35654SRS050026\_contig\_number\_10430SRS050422\_contig\_number\_33706SRS050925\_contig\_number\_25994SRS051610\_contig\_number\_9963SRS051882\_contig\_number\_21018SRS052697\_contig\_number\_25543SRS053214\_contig\_number\_5852SRS053398\_contig\_number\_12127SRS054905\_contig\_number\_20643SRS055017\_contig\_number\_22144SRS055982\_contig\_number\_11919SRS058770\_contig\_number\_23124SRS063040\_contig\_number\_36148SRS063190\_contig\_number\_10518SRS063518\_contig\_number\_33288SRS063985\_contig\_number\_contig-100\_170.166913SRS064557\_contig\_number\_20059SRS064645\_contig\_number\_1549SRS064757\_contig\_number\_20266SRS065504\_contig\_number\_12187SRS075078\_contig\_number\_21062SRS075773\_contig\_number\_31808SRS076756\_contig\_number\_6500SRS076804\_contig\_number\_16579SRS076929\_contig\_number\_contig-100\_226.144225SRS077127\_contig\_number\_12085SRS077194\_contig\_number\_10307SRS077392\_contig\_number\_30168SRS077552\_contig\_number\_21716SRS077641\_contig\_number\_14294SRS077730\_contig\_number\_10355SRS077849\_contig\_number\_23157SRS078176\_contig\_number\_18196SRS078242\_contig\_number\_19190SRS078419\_contig\_number\_22693SRS078665\_contig\_number\_9960SRS097958\_contig\_number\_21594SRS098073\_contig\_number\_6030SRS098881\_contig\_number\_15357SRS100021\_contig\_number\_8323SRS101376\_contig\_number\_689SRS101433\_contig\_number\_12254SRS103987\_contig\_number\_15803SRS1041037\_contig\_number\_3870SRS104311\_contig\_number\_26229SRS104636\_contig\_number\_29129SRS104975\_contig\_number\_9021SRS1054716\_contig\_number\_6618SRS1055022\_contig\_number\_6665SRS140492\_contig\_number\_contig-100\_120.202866SRS140513\_contig\_number\_11260SRS142503\_contig\_number\_39617SRS142890\_contig\_number\_23625SRS142923\_contig\_number\_8766SRS143181\_contig\_number\_5984SRS143417\_contig\_number\_3481SRS143876\_contig\_number\_16865SRS143895\_contig\_number\_10816SRS143991\_contig\_number\_23690SRS144183\_contig\_number\_17982SRS144297\_contig\_number\_6686SRS144362\_contig\_number\_10878SRS144506\_contig\_number\_23189SRS144603\_contig\_number\_309SRS146764\_contig\_number\_28523SRS147139\_contig\_number\_33527SRS147271\_contig\_number\_19420SRS147346\_contig\_number\_44891SRS147557\_contig\_number\_19313SRS148091\_contig\_number\_12708SRS148159\_contig\_number\_47637SRS148196\_contig\_number\_28592SRS148424\_contig\_number\_32579SRS148874\_contig\_number\_4058SRS149181\_contig\_number\_contig-100\_12.174686SRS149325\_contig\_number\_10180SRS892882\_contig\_number\_6834SRS893279\_contig\_number\_3878SRS893378\_contig\_number\_14614SRS893383\_contig\_number\_contig-100\_826.134447SRS971275\_contig\_number\_13006
28118
species

species
1638778

SRS045713\_contig\_number\_9105SRS051031\_contig\_number\_17911SRS063370\_contig\_number\_12145SRS077294\_contig\_number\_5412SRS143598\_contig\_number\_18628SRS893252\_contig\_number\_14285SRS893341\_contig\_number\_14794
7
